# Towards Real-Time Analysis of Gas-Liquid Pipe Flow: A Wire-Mesh Sensor for Industrial Applications

**DOI:** 10.3390/s23084067

**Published:** 2023-04-18

**Authors:** Philipp Wiedemann, Felipe de Assis Dias, Manuel Trepte, Eckhard Schleicher, Uwe Hampel

**Affiliations:** 1 Institute of Fluid Dynamics, Helmholtz-Zentrum Dresden-Rossendorf, Bautzner Landstraße 400, 01328 Dresden, Germany; 2 Teletronic Rossendorf GmbH, Bautzener Landstraße 45, 01454 Radeberg, Germany; 3 Chair of Imaging Techniques in Energy and Process Engineering, Technische Universität Dresden, 01062 Dresden, Germany

**Keywords:** wire-mesh sensor, two-phase flow, flow pattern identification, online data processing

## Abstract

Real-time monitoring of gas-liquid pipe flow is highly demanded in industrial processes in the chemical and power engineering sectors. Therefore, the present contribution describes the novel design of a robust wire-mesh sensor with an integrated data processing unit. The developed device features a sensor body for industrial conditions of up to 400 °C and 135 bar as well as real-time processing of measured data, including phase fraction calculation, temperature compensation and flow pattern identification. Furthermore, user interfaces are included via a display and 4…20 mA connectivity for the integration into industrial process control systems. In the second part of the contribution, we describe the experimental verification of the main functionalities of the developed system. Firstly, the calculation of cross-sectionally averaged phase fractions along with temperature compensation was tested. Considering temperature drifts of up to 55 K, an average deviation of 3.9% across the full range of the phase fraction was found by comparison against image references from camera recordings. Secondly, the automatic flow pattern identification was tested in an air–water two-phase flow loop. The results reveal reasonable agreement with well-established flow pattern maps for both horizontal and vertical pipe orientations. The present results indicate that all prerequisites for an application in industrial environments in the near future are fulfilled.

## 1. Introduction

In many industrial processes, e.g., oil and gas production, the chemical and pharmaceutical industries, food processing and power generation, multiphase flows of gas and liquids occur in manifold different scenarios and conditions. The knowledge and understanding of liquid and gas holdup, phase distributions, fluid velocities and flow morphologies are thus often key parameters for precise metering and intelligent process control. Moreover, they are mandatory for process intensification and sometimes important for safe plant operation, e.g., in the case of slug development in pipelines.

Nevertheless, there are only a few multiphase flow sensor systems available on the market for industrial process control. Mainly, such systems have been developed for monitoring and metering of oil–gas–water mixtures for the oil and gas industry. A recent overview can be found in [1]. In the past, the majority of such systems have utilized different mixing procedures to homogenize the fluid mixture in order to gather spatially and temporally averaged data of the flow composition as well as the mixture flow velocity. More recent developments tend towards fast tomographic sensors for industrial process control, cf. [2]. While fast tomographic X-ray scanners are still very expensive and suffer from radiation safety issues, electrical-impedance-based systems such as EIT and ECT have insufficient spatial resolutions. Among different tomographic techniques for multiphase flow measurement, wire-mesh sensors (WMSs), first introduced by Prasser et al. [3], have a high spatial resolution of typically 2…4 mm and a very high frame rate of up to 10,000 fps. The biggest drawback of WMSs is their intrusive character [4,5,6,7], which limits their application to processes and flow scenarios with no large particles streaming with the fluid mixture in order to prevent electrode destruction or sensor clogging. Additionally, WMSs so far have always used high-speed data recording with offline analysis afterwards, being unsuitable for online process control. Thus, during the last two decades WMSs have mainly been utilized in experimental test facilities emulating different kinds of industrial applications, such as steam generation in thermal power plants [8], pipe flows [9,10], chemical reactors [11], thermal separation units [12,13], etc. Even if the sensor is of intrusive nature, there is still a large field of real industrial processes being suitable for the application of WMS technology—in case it can become a robust 24/7 sensor system and it overcomes offline data processing. As one example, in direct steam generating solar power plants, there is a need for metering the amount of generated steam along the absorber tube system as well as for flow regime identification to control the system efficiently, cf. [14].

A first concept of a WMS targeting industrial applications was proposed in [15]. This sensor was equipped with an additional electronics unit, which controls the data acquisition and performs internal data reduction. More precisely, the calculation of cross-sectionally averaged phase fraction and the identification of flow patterns in the vertical upward gas–liquid flow were implemented. The achievements in [15] act as basis for our present work, which aims at providing an industrial type of wire-mesh sensor (indWMS) that is applicable to a much wider range of processes as well as to variable process conditions. Therefore, the newly developed indWMS provides a robust sensor body and electronics housing along with a redesigned electronics unit that allows for almost real-time data processing, including internal temperature compensation and data reduction by calculation of cross-sectionally averaged phase fractions and automatic flow pattern identification for vertical and horizontal pipe orientation. Moreover, a contemporary user interface and an automatic calibration routine were implemented for maximum user comfort. The developed device is described in detail in Section 2. Verification experiments and their results are presented in Section 3.

## 2. Design of an Industrial Wire-Mesh Sensor

### 2.1. Design of a Robust Sensor Body

**Figure 1:**
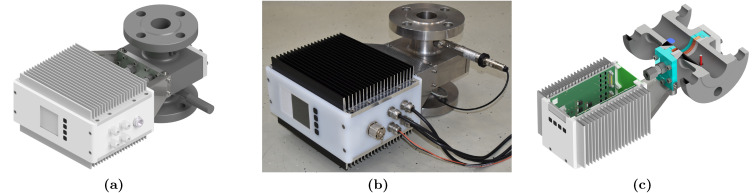
Industrial type of wire-mesh sensor: (**a**) 3D-CAD visualization of the sensor design; (**b**) photograph of the prototype; (**c**) sectional view.

While many experimental test facilities for multiphase flows are operated with air and water under ambient conditions, the industrial type of wire-mesh sensors has been designed to withstand pressures of up to 135 bar and temperatures of up to 400 °C for maximum applicability in practical processes, e.g., in solar thermal power plants, feed pipes of distillation columns, pipe lines, etc. The sensor system consists of a stainless-steel body flanged into the pipe at the position of interest and a sensor slot-in unit, as proposed in [16]. This design allows an easy exchange or maintenance of the sensor unit without dismantling the facility and decouples the purpose of electrode insulation and pressure barrier from each other. The slot-in sensor consists of a stainless-steel frame, which seals against the sensor body via a graphite gasket and a ceramic inlay accommodating the individually spring-mounted wire electrodes, cf. [17,18]. Upstream and downstream the slot-in unit a pressure and a temperature sensor are integrated into the system, respectively. The single stainless-steel electrodes run out of the sensor body via special eight-channel glands for pressure sealing and into a newly designed IP56-housing accommodating the electronics with its user interface via a 3.6-inch display with 4…20 mA connectivity for data transfer to any DAQ or real-time controller and an additional Ethernet interface for debugging and firmware updates. In order to achieve water tightness (IP56), the system is passively cooled by means of large aluminum heat spreaders. Visualizations of the sensor system are depicted in Figure 1.

### 2.2. Electronics Design

**Figure 2:**
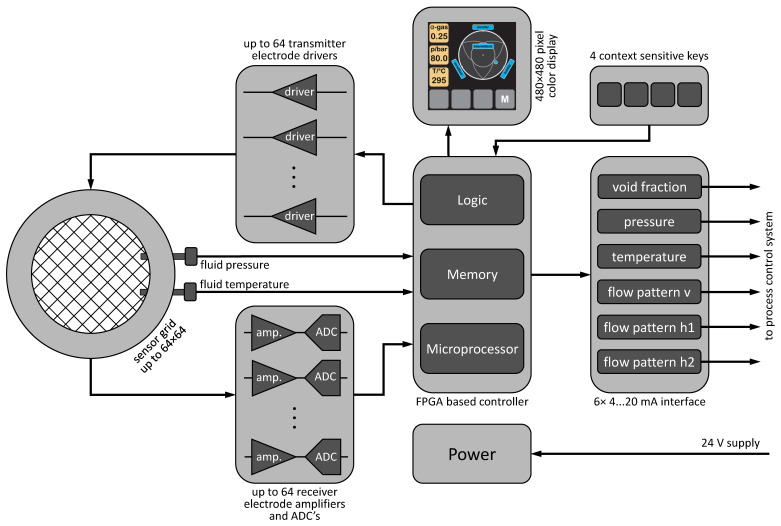
Block diagram of the electronic unit.

The electronic unit of the indWMS system consists of a FPGA-based controller unit, the transmitter and receiver blocks, an output driver section and the user interface. A block diagram of the new electronic system is shown in Figure 2.

The sensor grid itself comprises two planes of up to 64 parallel wire electrodes spanned with an equal distance over the cross-section of the pipe. The two electrode planes, transmitter and receiver plane, respectively, are oriented 90 to each other and have a small axial gap. The transmitter electrode drivers send a single bipolar voltage pulse of 6 µs length to each of the transmitter electrodes subsequently while the inactive transmitter electrodes are kept on ground potential. The receiver electrode amplifiers transform the resulting currents through the fluid in each of the receiver electrodes into a fully parallel voltage and convert it into a 16-bit digital value. After the last transmitter electrode is activated, the next frame starts. The frame rate of the measurement is currently limited to 5 kHz (for 16 transmitters). The controller unit is realized in an FPGA, which contains the programmable logic for parallel data processing in a pipeline architecture, the memory and a microprocessor for higher-level algorithms and communication protocols. This central controller unit realizes the synchronized timing of the transmitter and receiver unit. It also controls the user interaction via a 3.6-inch color display and a four-button context-sensitive keyboard. The display unit presents the measured and calculated parameters, instantaneous cross-sectional void fraction, pressure and temperature, and gives a graphical visualization of the identified flow pattern. It also shows the current menu functions for user access to set up the system.

The local instantaneous void fractions are calculated as averaged values over a period of one second. A ring buffer with a length of 64 s is utilized to derive the flow patterns instantaneously by using a fuzzy identification algorithm, cf. Section 2.3.2. The 4…20 mA interface can transfer all measured and calculated parameters to an external process control system. The identified flow regimes in vertical orientation are coded by means of a single value in a linear 4…20 mA scale, while in horizontal scenarios two 4…20 mA signals are necessary to transfer the polar coordinates representing the regime.

The system also includes a type K thermocouple as the temperature sensor, which is primarily utilized in the context of temperature compensation, cf. Section 2.3.1. Along with an additional pressure transducer, the local state variables at the position of the indWMS are provided and allow for external calculation of the corresponding thermodynamic properties of the fluids. Furthermore, alerts are generated on the basis of these measurements as soon as a predefined temperature or pressure is reached, cf. Section 2.4.

The power unit is based on a wide range 10…35 VDC DC-DC converter supplying all the required voltage levels of the system components.

### 2.3. Data Processing

#### 2.3.1. Temperature Compensation and Calculation of Cross-Sectionally Averaged Phase Fraction

The measurement principles of wire-mesh sensors rely on discriminating phases by differences in their electrical properties, cf. [3,19,20,21]. In the current version of the indWMS, the conductivity-based measurement principle according to [3] was implemented so far, focusing initially on gas–liquid flows with at least one conductive phase (water), which are encountered in many industrial applications, e.g., generation of process steam or water–steam cycles in power plants. In the conductivity-based approach, local (i,j) instantaneous (k) phase fractions $\alpha_{i,j,k}$ are usually quantified by normalizing the measured voltage signals $U_{i,j,k}^{meas}$, which represent the conductivity distribution in the pipe’s cross-section with respect to a single-phase reference measurement of liquid $U_{i,j}^{liquid}$. However, since the electrical conductivity of water is known to exhibit a strong temperature dependence, the temperatures of reference and two-phase measurement need to coincide. This may be achieved straightforwardly in most lab applications by recording several reference measurements if temperature drifts occur. In contrast, this is seldom possible in most industrial processes, since flexible operations with continuous transitions between partial load and overload scenarios would lead to a tremendous effort for reference measurements only. Furthermore—and much more seriously—pure single-phase liquid flow is hardly obtained at saturation temperature, which is usually the region of interest for two-phase flow measurements. Therefore, the temperature compensation method proposed by [22] was implemented in the real-time data processing unit of the indWMS system. The adapted formulation for the quantification of phase fractions reads

(1) $$\alpha_{i,j,k}^{gas}\left(T\right)=1-\frac{U_{i,j,k}^{meas}\left(T\right)}{F\left(T,T_{ref}\right)\;U_{i,j}^{liquid}\left(T_{ref}\right)}.$$

Here, F denotes the temperature compensation factor that is calculated on the basis of the ISO 7888 model [23] using the temperatures of the reference and two-phase measurement, $T_{ref}$ and T, respectively, cf. [22]. Hence, the single-phase reference matrix $U_{i,j}^{liquid}$ recorded at an arbitrary reference temperature $T_{ref}$ can be converted to a reference matrix at temperature T corresponding to the present conditions of the two-phase measurement $U_{i,j,k}^{meas}$. A prerequisite for applying Equation (1) is that the gain settings of the transimpedance amplifiers are identical for the reference and the two-phase measurement. Details on adjusting the amplifiers are given in Section 2.4.

All values of $\alpha_{i,j,k}^{gas}$ are subsequently limited to the interval $\left[0\ldots 1\right]$. Eventually, the cross-sectional average is calculated as

(2) $$\alpha_{k}^{gas}=\underset{i}{\sum }\underset{j}{\sum }a_{i,j}\;\alpha_{i,j,k}^{gas},$$

with $a_{i,j}$ denoting the share of a pixel i,j with the pipe’s cross-section, cf. [15]. For the output via display and 4…20 mA signal, a temporal average $\alpha^{gas}$ is calculated from the last k=1…5000 frames and updated every second.

#### 2.3.2. Flow Pattern Identification

Algorithms for flow pattern identification were implemented for vertical and horizontal pipe orientations according to [15] and [24], respectively. Both algorithms are based on the analysis of statistical features of the measured three-dimensional phase fraction distributions $\alpha_{i,j,k}^{gas}$ and provide the final identification result in a fuzzy manner. Based on a so-called degree of membership, the output describes the likelihood of a certain measurement to belong to one or more predefined flow patterns. According to [25], four major flow patterns were selected for each pipe orientation, namely bubbly, slug, churn and annular flow for vertical and bubbly, intermittent, stratified and annular flow for horizontal pipe orientation. The identification algorithms use a time span of 64 s as input from the ring buffer and the result is updated every second. In this way, transitions of flow patterns can also be captured almost in real time. The results of the flow pattern identification are represented graphically on the display (see Section 2.4) and are coded as 4…20 mA output signals as well (cf. Section 2.2).

### 2.4. User Interface

**Figure 3:**
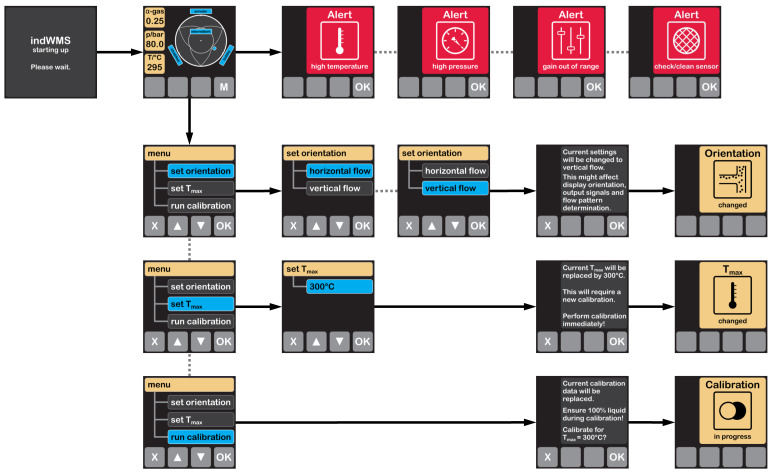
Display screens and menu structure of the indWMS.

The user interface with display and context-sensitive keys was integrated to provide instant information about the present flow conditions and the sensor status as well as to configure the sensor during the commissioning process. After switching on the power supply, a boot screen is shown temporarily. Subsequently, the sensor system directly activates the measurement protocol and switches to the main screen, in which current data of temperature, pressure and cross-sectional gas phase fraction are displayed as numerical values, cf. Figure 3. The identified flow pattern is visualized in a fuzzy manner, as proposed by [15] and [24] for vertical and horizontal sensor positions, respectively. Additionally, alerts are displayed automatically, if certain events occur, cf. Section 2.2 and Figure 3. Using the keys next to the display, the user can switch to and navigate through the configuration menu. Here, three configurations are available and need to be set when putting the sensor into operation:

1. Switching between horizontal and vertical orientations leads to activation of the respective flow pattern identification algorithm and its visualization scheme on the main screen as well as to a change in the display orientation.
2. Adjusting the expected maximum temperature of the two-phase flow is required for the calibration routine, which is explained below.
3. Running the calibration routine.

**Figure 4:**
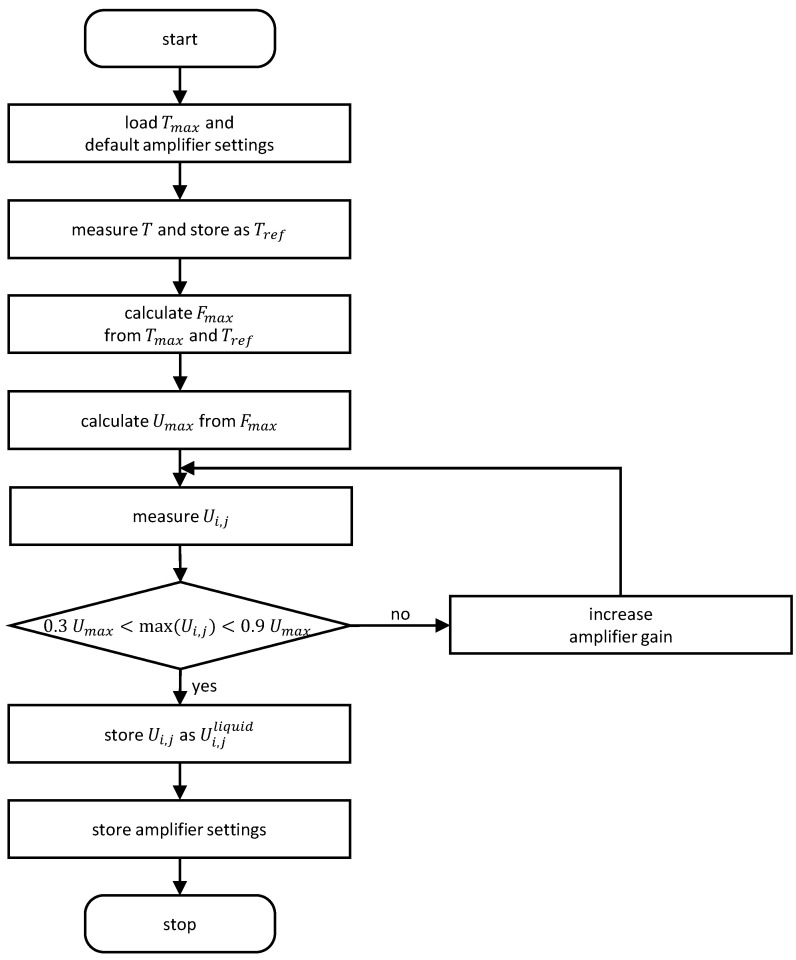
Flow chart for autonomous adjustment of amplifier gains and acquisition of the reference data set.

Running the calibration routine basically means that the current flow conditions are treated as the reference state ($T_{ref}\leftarrow T$) and that the reference matrix $U_{i,j}^{liquid}\left(T_{ref}\right)$ of the completely liquid-filled pipe is recorded. However, since the indWMS accounts for temperature compensation in order to provide reliable measurements over a wide range of operating conditions, the amplifier gains in the receiver circuit need to be adjusted in an anticipatory manner at the calibration stage already. More precisely, the amplifier gains must be configured to meet an optimal range of signal response, e.g., to avoid overdrive, at the targeted operating temperature, or rather the corresponding electrical conductivity of the liquid. As the absolute electrical conductivity and its temperature-induced change is assumed to be unknown in most applications, an iterative procedure was implemented in the indWMS system to allow for an autonomous adjustment of amplifier gains. Here, firstly the relative change in the electrical conductivity is estimated by a maximum compensation factor $F_{max}\left(T_{max},T_{ref}\right)$, which is based on the user-defined maximum temperature $T_{max}$ of the two-phase process and the one of the present reference state $T_{ref}$. As $F_{max}$ applies to both conductivity and the measured voltage signal in the receivers, cf. [22], a maximum permissible voltage $U_{max}$ can be estimated from the total ADC range. Subsequently, reference data are acquired and compared against $U_{max}$. Based on the result, the amplifier settings may become modified until agreement is obtained with a defined tolerance range. The flow chart of the calibration routine is depicted in Figure 4.

## 3. Experimental Verification

### 3.1. Temperature Compensation

#### 3.1.1. Experimental Procedure

**Figure 5:**
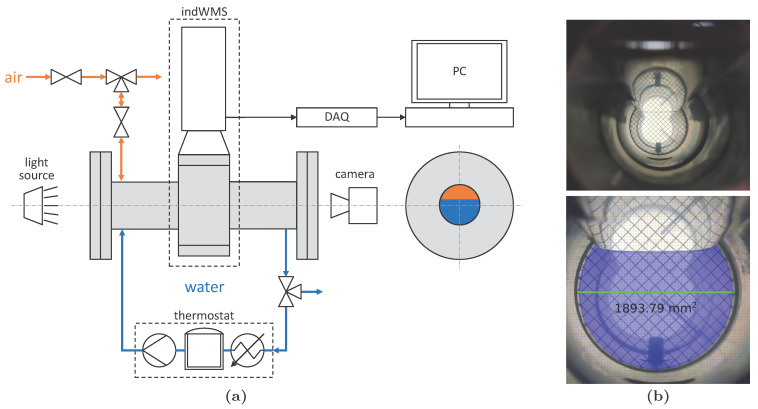
(**a**) Experimental setup for verifying phase fraction measurement combined with temperature compensation; (**b**) images of pipe cross-section recorded by high-resolution camera: (**top**) raw image, (**bottom**) cropped and scaled image with calculation of $A_{liquid}$.

To verify the implemented temperature compensation algorithm, the cross-sectionally averaged phase fractions, which are measured by the indWMS at different temperatures, need to be compared against reference data from a temperature-independent measurement technique. For that purpose, the experimental setup shown in Figure 5a was utilized. Here, the indWMS was mounted horizontally between flanges with transparent inspection glasses, allowing for visual observation of the measurement plane. Tap water with a conductivity of $\kappa_{25{}^{\circ}C}=395\;µS/\mathrm{cm}$ was circulated through the system at defined temperatures by means of a Lauda ProLine RP870 thermostat. In a first step, the pipe was filled with liquid of 24 °C completely and the start-up routine of the indWMS, i.e., autonomous adjustment of amplifier settings and reference measurement (cf. Section 2.3.1 and Section 2.4), was performed. Then, the liquid temperature was increased step-wise with the thermostat. After a stable temperature was obtained in the sensor at each temperature level, different filling heights of liquid were adjusted by injecting a gas layer of pressurized air. This procedure artificially emulates a two-phase condition below the actual saturation temperature of the liquid. At the same time, the liquid flow was paused for ensuring a smooth gas–liquid interface. For each set of temperature level and filling height, the 4…20 mA signals of the indWMS were recorded for at least one minute with a sampling frequency of 2 Hz using a National Instruments CompactDAQ Chassis and a PC. In parallel, a photo of the sensor’s measurement plane was recorded against backlight using a 48 mega pixel camera. The photos had a resolution of approximately 25 px/mm in the area of interest and served as a basis for evaluating the indWMS data.

.

In order to provide numerical values from the image references, further processing was accomplished using the graphics software Inkscape v0.92. The following steps were conducted manually for each individual measurement point:

1. Image cropping;
2. Image scaling according to the pipe’s inner diameter (green line in Figure 5b);
3. Approximating the gas–liquid interface in the stratified state within a concentric circle of the pipe’s inner diameter (taking menisci at the pipe wall into consideration);
4. Calculating the area $A_{gas}$ or $A_{liquid}$ of the remaining geometry (blue area in Figure 5b).

Finally, the cross-sectional phase fraction was calculated as follows:

(3) $$〈\alpha^{gas}〉=\frac{A_{gas}}{A_{pipe}}=1-\frac{A_{liquid}}{A_{pipe}}\;\;.$$

The absolute uncertainty of $〈\alpha^{gas}〉$ from image processing is estimated to be lower than 0.01.

For the cross-sectional phase fractions measured by the indWMS, temporal averages of the time series are reported in the following section. The averaging periods were chosen in a way to exclude noticeable temperature drops that particularly occurred at higher temperature levels. In this way, a maximum uncertainty of 1.8 K was achieved for the temperature measurements. The relative uncertainty of phase fraction measurements with wire-mesh sensors was assessed with 10.5% by Tompkins et al. [6] by comparison with other measurement techniques and accounting for a large variety of complex flow patterns. However, much lower uncertainty can be expected here, since all measurements are conducted under an ideal stratified condition and steady state.

#### 3.1.2. Results and Discussion

**Figure 6:**
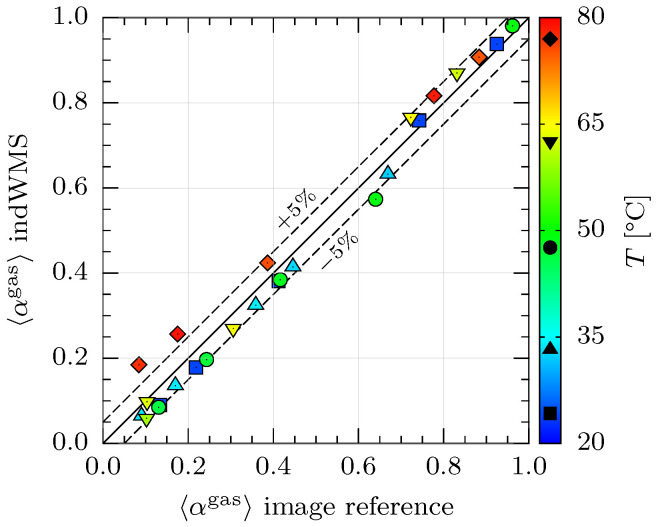
Comparison of cross-sectional gas phase fractions $〈\alpha^{gas}〉$.

The cross-sectional gas phase fractions that were obtained at various temperature levels from the indWMS and the temperature-independent image reference are compared in Figure 6. Since the majority of the measurements exhibit an absolute deviation of less than ±5% while the electrical conductivity of water and thus the measured voltage signals are expected to increase by a factor of up to 2.5 in the investigated temperature range, cf. [22], it can be concluded that the implemented temperature compensation works excellently.

All minor discrepancies that are observed for measurements of up to 65 °C, i.e., a temperature drift of 41 K, are assumed independent of the temperature compensation method, since they show the identical trend when compared to the results of the measurements at the reference temperature of 24 °C. For high liquid levels, i.e., low $〈\alpha^{gas}〉$, some pixels on the top of the pipe were observed to exhibit a signal indicating liquid, despite being located in the stratified gas zone. Consequently, slightly lower $〈\alpha^{gas}〉$ are obtained from the indWMS here. As no water was observed visually in that region, we suppose an influence of the wire fixations in the sensor slot-in unit. On the other side, positive deviations of the indWMS at very low liquid levels, i.e., high $〈\alpha^{gas}〉$, might be related to the intrusive nature of the measurement technique. Here, liquid lamellas were observed visually and in the images between the pipe wall and the outer wires above the horizontal gas–liquid interface. Since they are considered analogously to menisci in the image reference (cf. Figure 5b), along with the uncertainty of the 2D projection regarding whether they actually stick to both wire planes and are consequently measured as liquid, overestimation of $〈\alpha^{gas}〉$ can be explained.

In contrast to the above descriptions, obviously larger and exclusively positive deviations of up to +10% are observed at a temperature level of 80 °C. As the implemented temperature compensation algorithm has proven to work satisfactorily for temperature drifts of up to 60 K in single-phase flow (according to [22]), the increased deviations in Figure 6 must be explained by the gradual onset of degassing or boiling. Due to locally higher temperatures in the heating section of the thermostat, micro-bubbles of gas or vapor are formed and transported to the test section inside the continuous liquid phase. This phenomenon was also observed visually, but is not captured in the photos nor the image-processing procedure. However, the indWMS data reflect the visual observation by higher gas fractions. The effect is more dominant at higher liquid levels, i.e., low $〈\alpha^{gas}〉$, due to a higher share of the affected cross-sectional area. It can be stated that the increased deviations at the temperature level of 80 °C originate from the experimental setup and procedure, while the temperature compensation is assumed to work properly. The above finding also shows that a sufficient distance needs to be kept between $T_{ref}$ and $T=T_{sat}$ when running the calibration routine in practical applications.

In summary, it can be concluded that the implemented algorithms for temperature compensation and phase fraction calculation work satisfactorily. Temperature drifts of up to 55 K can be compensated with an average deviation of only 3.9% across the full range of phase fractions. However, improvement of the wire fixations is necessary to recover the desired accuracy. Moreover, transient analysis is needed in future studies to determine the signal response characteristics related to thermal inertia of the system, particularly the thermocouple used for the temperature measurement.

### 3.2. Real-Time Flow Pattern Identification

#### 3.2.1. Experimental Procedure

Investigations for the verification of the flow pattern identification algorithms were performed using the experimental facility described in [24]. While the indWMS was installed at 75 L/D downstream the gas injection for horizontal flows, only 36 L/D were feasible for vertical pipe orientation due to height limitations. The experiments and data acquisition were conducted according to [24]. Several measurements were performed at different combinations of gas and liquid flow rates to cover all possible flow patterns and phase fractions. Measurement uncertainty with respect to the resulting superficial velocities is presented in [24]. At each measurement point data were sampled over 180 s with the indWMS to allow for three cycles of data buffering. Since the flow pattern remained constant at each measurement point and the signals of the indWMS showed negligible fluctuations, temporal averages are reported in the following evaluation.

#### 3.2.2. Results of Cross-Sectional Phase Fraction and Flow Pattern Identification in Vertical Pipes

**Figure 7:**
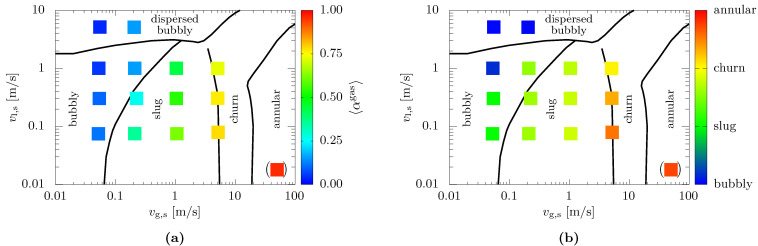
Comparison of measurement results from indWMS against Barnea’s [26] flow pattern map for vertical pipe orientation: (**a**) cross-sectionally averaged gas fraction; (**b**) flow pattern.

The measurement results of the indWMS are plotted against the flow pattern map of Barnea [26] in Figure 7. Note that the bracketed point in the annular regime is not related to the readable superficial velocities, but was produced artificially by stagnant gas and a falling liquid film. Figure 7a shows that the cross-sectional gas fractions have both plausible values and comprehensible trends when compared to the orientation of the axes and the expectations derived from the flow pattern map. Moreover, reasonable quantitative agreement can be attested for the comparison of the measured gas fractions with experimental data from the literature (refer to Jones and Zuber [27] and Rosa et al. [28]).

With regard to the flow patterns, Figure 7b shows that the identification results from the indWMS agree fairly well with the predictions of Barnea’s map [26]. However, premature transitions of the measured flow patterns are observed when increasing the superficial gas velocity, particularly at low liquid flow rates. This effect might be related to the relatively short distance between gas injection and measurement position impeding full development of the flow.

#### 3.2.3. Results of Cross-Sectional Phase Fraction and Flow Pattern Identification in Horizontal Pipes

**Figure 8:**
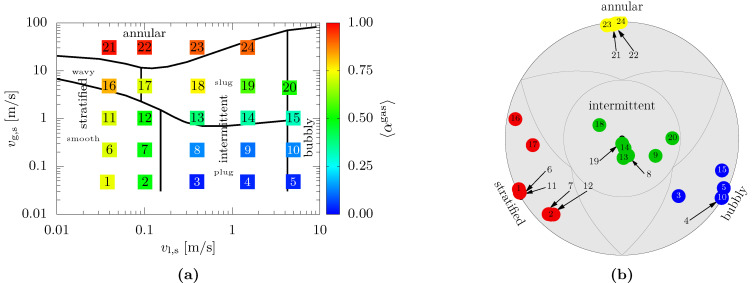
Comparison of measurement results from indWMS against Mandhane’s [29] flow pattern map for horizontal pipe orientation: (**a**) cross-sectionally averaged gas fraction; (**b**) flow pattern in terms of polar coordinates according to [24] (color indicates most dominant membership).

The results of the indWMS measurements in the horizontal pipe are depicted in Figure 8 along with the flow pattern map of Mandhane [29]. In analogy to the vertical pipe orientation, the cross-sectional gas fractions in Figure 8a show plausible values and comprehensible trends when compared to the orientation of the axes and the expectations derived from the flow pattern map again. A quantitative comparison against the experimental data in [24] reveals excellent agreement of the phase fraction distribution among the map, which is, however, expected due to the high reproducibility of the test conditions with the utilized experimental setup.

The flow patterns identified by the indWMS are depicted in Figure 8b using the polar representation proposed by [24]. The numbers refer to the measurement points in Figure 8a to allow for comparison against the flow pattern map of Mandhane [29]. It can be seen that excellent agreement is obtained for the present measurements. In particular, transitional regimes are captured well by the fuzzy methodology, e.g., point 16 (stratified/annular), point 17 (stratified/intermittent/annular) and point 20 (intermittent/bubbly). With regard to points 3 and 4, which appear to be identified incorrectly by the indWMS, we need to point out that plug flow at low gas velocities features very small gas pockets only (see $〈\alpha^{gas}〉$ in Figure 8a also) and is thus sometimes called elongated bubble flow [30] or bubbly-transitional flow [31] in the literature. In contrast to this subjective interpretation (see also discussions in [24,32]), the objective differentiation by structural analysis of the phase distribution of the indWMS is assumed to be more reliable here.

Finally, it can be concluded that the new indWMS system is able to identify flow patterns in vertical as well as horizontal pipes satisfactorily. However, further quantitative validation against references from other measurement techniques is necessary.

## 4. Conclusions

An industrial type of wire-mesh sensor with novel real-time data processing and a user interface was developed and tested successfully. It was designed for high-temperature and high-pressure applications and has three main functionalities: (1) temperature compensation, (2) calculation of cross-sectional phase fraction and (3) real-time flow pattern identification. All these functionalities were demonstrated experimentally for air–water two-phase flows. In the first demonstration, cross-sectionally averaged phase fractions along with temperature compensation were evaluated. The experimental results show that temperature drifts of up to 55 K can be compensated, leading to average deviations of only 3.9% across the full range of phase fractions. Eliminating shortcomings with the constructive design of the wire fixation will lead to higher accuracy in the future. The automatic flow pattern identification was verified in an air–water two-phase flow loop, showing reasonable agreement with flow pattern maps from the literature for horizontal and vertical pipe orientation. Further work with the developed indWMS system will focus on three aspects: (1) quantitative validation of flow pattern measurement by comparison with other tomographic techniques, (2) study of dynamic flow scenarios, i.e., temporal evolution of temperature and/or flow pattern, as well as the response behavior of the developed system, and finally, (3) evaluation of the indWMS in real industrial applications. In addition, we aim at the implementation of the capacitive and dual modality measurement principles, higher frame rates and advanced data processing using GPUs in the future.

## Data Availability

The experimental data presented in this study are available at doi:10.14278/rodare.2182 upon reasonable request.
